# The Coffee Diterpene, Kahweol, Ameliorates Pancreatic β-Cell Function in Streptozotocin (STZ)-Treated Rat INS-1 Cells through NF-kB and p-AKT/Bcl-2 Pathways

**DOI:** 10.3390/molecules26175167

**Published:** 2021-08-26

**Authors:** Waseem El-Huneidi, Shabana Anjum, Khuloud Bajbouj, Eman Abu-Gharbieh, Jalal Taneera

**Affiliations:** 1 Department of Basic Medical Sciences, College of Medicine, University of Sharjah, Sharjah 27272, United Arab Emirates; Kbajbouj@sharjah.ac.ae; 2 Sharjah Institute for Medical Research, University of Sharjah, Sharjah 27272, United Arab Emirates; anjum6repro@gmail.com (S.A.); eabugharbieh@sharjah.ac.ae (E.A.-G.); 3 Department of Clinical Sciences, College of Medicine, University of Sharjah, Sharjah 27272, United Arab Emirates

**Keywords:** kahweol, diabetes, streptozotocin, apoptosis, pancreatic β-cells, insulin secretion, INS-1 cells

## Abstract

Kahweol is a diterpene molecule found in coffee that exhibits a wide range of biological activity, including anti-inflammatory and anticancer properties. However, the impact of kahweol on pancreatic β-cells is not known. Herein, by using clonal rat INS-1 (832/13) cells, we performed several functional experiments including; cell viability, apoptosis analysis, insulin secretion and glucose uptake measurements, reactive oxygen species (ROS) production, as well as western blotting analysis to investigate the potential role of kahweol pre-treatment on damage induced by streptozotocin (STZ) treatment. INS-1 cells pre-incubated with different concentrations of kahweol (2.5 and 5 µM) for 24 h, then exposed to STZ (3 mmol/L) for 3 h reversed the STZ-induced effect on cell viability, apoptosis, insulin content, and secretion in addition to glucose uptake and ROS production. Furthermore, Western blot analysis showed that kahweol downregulated STZ-induced nuclear factor kappa B (NF-κB), and the antioxidant proteins, Heme Oxygenase-1 (HMOX-1), and Inhibitor of DNA binding and cell differentiation (Id) proteins (ID1, ID3) while upregulated protein expression of insulin (INS), p-AKT and B-cell lymphoma 2 (BCL-2). In conclusion, our study suggested that kahweol has anti-diabetic properties on pancreatic β-cells by suppressing STZ induced apoptosis, increasing insulin secretion and glucose uptake. Targeting NF-κB, p-AKT, and BCL-2 in addition to antioxidant proteins ID1, ID3, and HMOX-1 are possible implicated mechanisms.

## 1. Introduction

Diabetes mellitus (DM) is a metabolic condition associated with high blood glucose levels over long periods. The most common types of DM are type 1 and type 2 diabetes. T1D is characterized by β-cell dysfunction and apoptosis due to autoimmune disorder, leading to a lifelong reliance on insulin therapy. On the other hand, T2D is characterized by insufficient insulin and/or insulin resistance [1]. Typically, secreted insulin from pancreatic β-cells maintain normal glucose concentration in circulation [1]. Consequently, dysfunctional conditions of pancreatic β-cells, such as apoptosis, glucotoxicity, and oxidative stress, may cause disturbance in insulin production and secretion [2].

Glucose molecules enter cells through glucose transporters under prolonged hyperglycemic conditions; consequently, an increasing amount of glycolysis occurs in the β-cells, culminating in a further increase in ROS (reactive oxygen species) production through multiple routes [3]. When ROS continuously increases, oxidative stress occurs, leading to cell damage and apoptosis in pancreatic cells. Therefore, inhibiting ROS formation from glucose metabolism may reduce oxidative damage and apoptosis in pancreatic cells [3].

**Figure 1:**
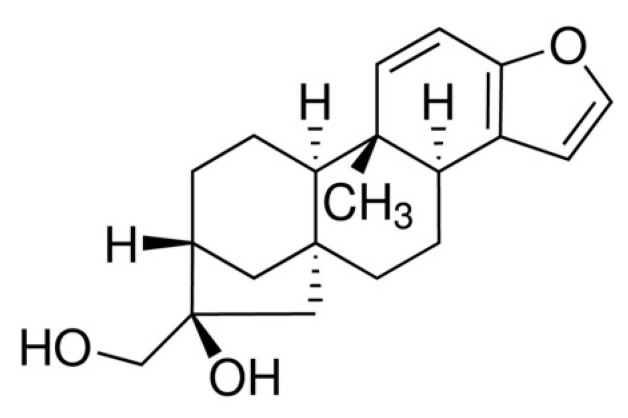
Chemical structure of kahweol.

A growing body of research suggests that a healthy lifestyle with exercise and diet can mitigate T2D. In addition, various epidemiological studies suggest that moderate coffee intake is allied with a lower risk of developing T2D [4,5]. Kahweol (as shown in Figure 1) is a coffee diterpene molecule found in abundance in arabica coffee bean oil [6]. In arabica coffee, kahweol content ranges between 661 and 923 mg/100 g [7], while in coffee oil, kahweol concentration is between 33 and 42 g/Kg oil [8]. Recently, it has been shown that kahweol has various physiological activities, including anti-proliferative, anti-inflammatory, anti-fibrotic, anti-angiogenic, and antioxidant effects in different cell types [9,10]. In addition, kahweol has been demonstrated to diminish liver inflammation in hepatocytes by reducing the activation of nuclear factor kappa B (NF-kB), as well as signal transducer and transcription factors (STAT3) [11].

Nonetheless, the issue of whether kahweol could have a protective role in β-cell damage is unknown. Therefore, the effect of kahweol pre-treatment on STZ-induced apoptosis and oxidative stress in rat insulinoma (INS-1 832/13) cells was examined in this work. Moreover, we explored the NF-κB signaling pathway as a possible mechanism. 

## 2. Results

### 2.1. Kahweol Protects INS-1 Cells against STZ-Induced Damage 

**Figure 2:**
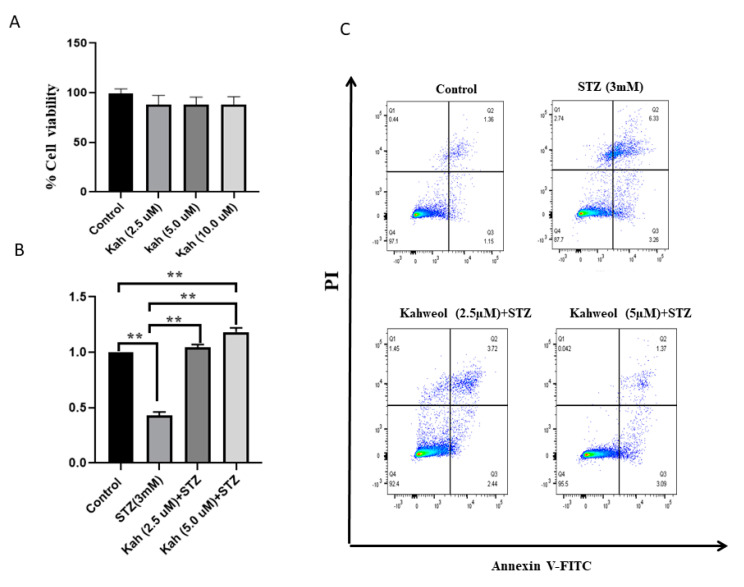
Effect of kahweol on viability and apoptosis in STZ-treated cells. (**A**) Percentage of cell viability in INS-1 cells treated with different concentrations of kahweol (2.5, 5, 10 µM) for 24 h compared to control cells (untreated cells). (**B**) Percentage of cell viability in INS-1 cells exposed to STZ (3 mM) or cells pre-incubated for 24 h with different concentrations of kahweol (2.5 and 5 µM) followed by STZ treatment compared to control cells. (**C**) Assessment of apoptosis in control cells, STZ-treated cells for 3 h and cells pre-incubated with kahweol for 24 h followed by STZ-treatment as analyzed by flow cytometry. Q1 represents the necrotic cell population, Q2 represents the late apoptosis cell population, Q3 represents the early apoptosis cell population. Data are shown from three independent experiments. ** *p* < 0.01. Bars represent mean ± SD.

As illustrated in Figure 2A, MTT assay results revealed that treating INS-1 cells with various concentrations (2.5–10 µM) of kahweol showed no significant effect of cell viability compared to untreated control cells (*p* > 0.05). In contrast, a substantial reduction in viable cells (~50%; *p* < 0.05) was observed in INS-1 cells exposed to STZ (3 µM) for 3 h (Figure 2B). Next, we tested whether kahweol has a protective effect in INS-1 against STZ-treatment. As shown in Figure 2C, cells pre-incubated with different concentrations (2.5 and 5.0 µM) of kahweol for 24 h prior to STZ-treatment exhibited a dramatic improvement in cell viability level (~55%; *p* < 0.05). 

Likewise, apoptosis assay tested by annexin-V staining revealed a significant increase (~9.5%; *p* > 0.05) in the percentage of apoptotic cells (early and late apoptosis) out of the total number of STZ-treated cells, compared to 2.4% in the control cells (Figure 2C, upper panels). Necrotic cells stained by the counterstain propidium iodide (PI) that discriminates necrotic cells from apoptotic cells showed a 2.7% in STZ-treated cells compared to 0.4% in the control cells (*p* > 0.05) (Figure 2C, upper panels). Interestingly, cells pre-incubated with 2.5 µM of kahweol showed a significant decrease (*p* > 0.05) in the apoptotic (6%) and necrotic cells (1.4%) compared to cells with no kahweol (Figure 2C, lower panels). The decrease in apoptotic (4.3%) and necrotic (0.04%) cells was greater with 5 µM kahweol treatment (*p* > 0.05) compared to STZ-treatment alone (Figure 2C, lower panels). These findings demonstrate that kahweol can protect INS-1 cells against STZ-induced damage. 

### 2.2. Kahweol Increases Insulin Secretion and Content in STZ-Treated Cells

**Figure 3:**
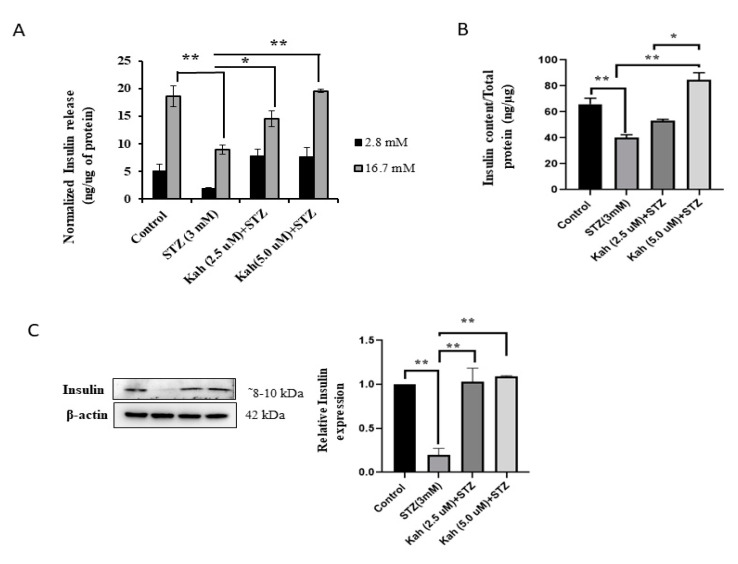
Effect of kahweol on insulin secretion, content, expression, and glucose uptake in STZ-treated cells. (**A**) Normalized stimulated insulin secretion in response to 2.8 mM glucose and 16.7 mM glucose as determined by ELISA in STZ-treated cells for 3 h or cells pre-incubated with kahweol for 24 h followed by STZ-treatment compared to control cells. (**B**) Normalized insulin content measurements from STZ-treated cells for 3 h or cells pre-incubated with kahweol for 24 h followed by STZ-treatment compared to control cells. (**C**) Western blot analysis of INS (insulin) in STZ-treated cells for 3 h or cells pre-incubated with kahweol for 24 h followed by STZ-treatment compared to control cells. β-actin was used as endogenous control. Data are shown from three independent experiments. * *p* < 0.05 and ** *p* < 0.01. Bars represent mean ± SD.

As illustrated in Figure 3A, INS-1 cells treated with STZ showed significant decrease in glucose-stimulated insulin secretion (GSIS) at basal level (2.8 mM) (~50%; *p* < 0.05) and stimulation level (16.7 mM) (~44%; *p* < 0.05) compared to control cells. Furthermore, STZ-treated cells showed less insulin content (~40%; *p* < 0.05) compared to untreated cells (Figure 3A).

Interestingly, pre-incubating STZ-treated INS-1 cells with kahweol (2.5 and 5 µM) for 24 h exhibited a significantly increased insulin secretion at 2.8 mM or 16.7 mM glucose (*p* < 0.05) (Figure 3A). A three-fold increase in basal insulin secretion was observed at 2.5 and 5 µM of kahweol pre-incubation. The stimulation of insulin secretion increased almost two-fold (Figure 3A) relative to STZ-treated cells. The increase in insulin secretion was associated with a dramatic elevation in insulin content in cells pre-incubated with kahweol compared to STZ-treated cells (*p* < 0.05) (Figure 3B). Cells pre-incubated with 5 µM of kahweol showed an almost one-fold increase in insulin content (Figure 3B). This was further confirmed with Western blot of insulin, where STZ-treated cells showed a significant decrease in insulin expression while kahweol treated cells recovered the insulin expression, as shown in Figure 3C.

### 2.3. Kahweol Reduces Intracellular ROS and Increases Glucose Uptake in INS-1 Cells

**Figure 4:**
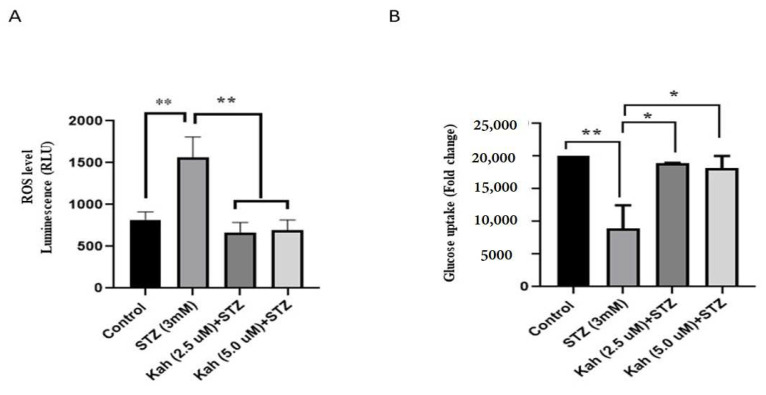
Effect of kahweol on ROS production and glucose uptake in STZ-treated cells. (**A**) Levels of ROS were determined by fluorescence intensity in STZ-treated cells for 3 h or pre-incubated with kahweol for 24 h, followed by STZ-treatment compared to control cells. (**B**) Glucose uptake assessment as determined by flow cytometry in STZ-treated cells for 3 h or pre-incubated with kahweol for 24 h followed by STZ-treatment compared to control cells. Data are shown from three independent experiments. * *p* < 0.05 and ** *p* < 0.01 Bars represent mean ± SD.

ROS measurements in STZ-treated cells revealed an increase in the ROS level luminescence (RLU) (~50%; *p* < 0.05) compared to control cells (Figure 4A). Alternatively, kahweol pre-incubated cells (2.5 or 5 µM) substantially reduced (~60%; *p* < 0.05) the ROS level luminescence (RLU) when compared to STZ-treated cells (Figure 4A). In addition, we found that STZ-treated cells exhibited a significant decrease in the level of glucose uptake (~60%; *p* < 0.05) relative to control cells (Figure 4B). Pre-incubated cells with kahweol (2.5 or 5 µM) reversed glucose uptake activity (~55%; *p* < 0.5) to average level compared to STZ-treated cells (Figure 4B).

### 2.4. Kahweol’s Effect on NF-κB Activation, p-AKT and Bcl-2, HMOX1, ID1 and ID3 Protein Expression

**Figure 5:**
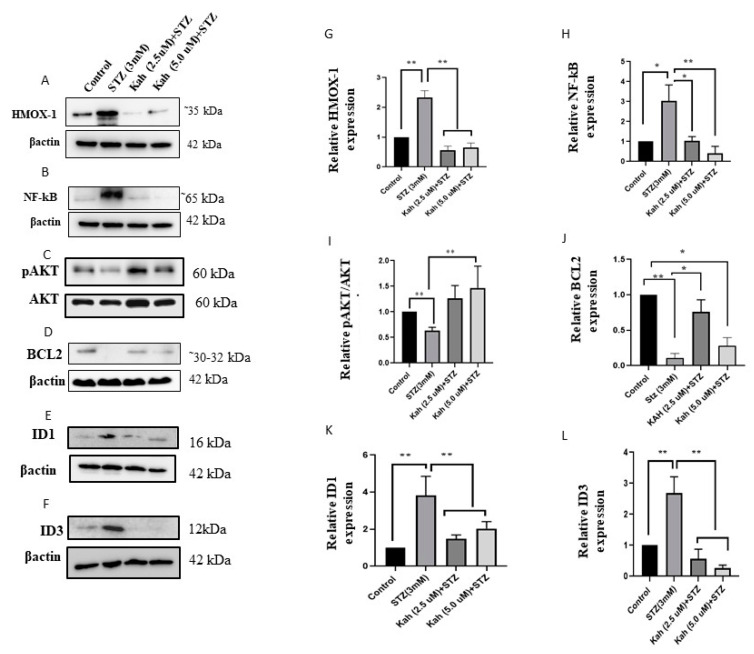
Effect of kahweol on the protein expression in STZ-treated cells. Western blot analysis of HMOX1 (**A**,**G**), NF-κB (**B**,**H**), p-AKT (**C**,**I**), Bcl2 (**D**,**J**), ID1 (**E**,**K**), and ID3 (**F**,**L**) in STZ-treated cells for 3 h or cells pre-incubated with kahweol for 24 h followed by STZ-treatment compared to control cells. β-actin was used as endogenous control. * *p* < 0.05 and ** *p* < 0.01. Bars represent mean ± SD.

As illustrated in Figure 5, STZ-treated cells induced the expression of H1 (Figure 5A,G) and NF-κB (Figure 5B,H), ID1 (Figure 5E,K), and ID3 (Figure 5F,L), whereas marked suppression of Bcl2 (Figure 5D,J) was observed. In addition, the relative Expression of p-AKT/AKT showed a significant reduction was observed (Figure 5C,I). Upon pre-incubation with 2.5 or 5 µM of kahweol, a significant down-regulation of NF-κB, Hmox1, ID1, and ID3 was manifested (*p* < 0.05). In contrast, the expression of BCL-2 and relative p-AKT/AKT was increased after kahweol pre-incubation (*p* < 0.05), as shown in Figure 5.

## 3. Discussion

Coffee is among the most extensively consumed drinks globally; it contains many effective anti-diabetic compounds, including caffeine, kahweol, cafestol, and chlorogenic acid [12,13,14,15,16,17,18]. On the other hand, literature reported that high coffee consumption is associated with some adverse effects such as gastrointestinal disturbances, tachycardia, and sleep disorders. However, these effects are mainly related to methylxanthine alkaloid, caffeine, especially when consumed orally in a dose of more than 400 mg per day [19]. On the other hand, cafestol and kahweol have been reported to be linked to lipid disturbances [10]. 

Yet, the anti-diabetic effect of kahweol has not been investigated. This study demonstrated that kahweol could protect the clonal rat pancreatic β-cell (INS-1832/13) against STZ-induced damage, enhanced insulin secretion, insulin content, decreased ROS levels, and increased glucose uptake. Moreover, we found that cells pre-incubated with kahweol down-regulated the expression of NF-κB, HMOX1, ID1, and ID3 and upregulated the expression of BCL-2, p-AKT, and INS. Thus, to the best of our knowledge, this is the first study that demonstrates a beneficial effect of kahweol on pancreatic β-cells.

Our data showed that kahweol restored the capacity of INS-1 cells to produce and secrete insulin and glucose uptake in the STZ-treated cells. In line with our finding, it has been reported that cafestol, a fatty ester present in Turkish coffee with a single bond in its structure, can stimulate glucose uptake and secretion of insulin in INS-1E and muscle cells [15]. In contrast, oxokahweol, a synthetic derivative of kahweol, showed no significant effects on insulin secretion [15]. Even though the impact of oxokahweol was unexpected, the molecular mechanism underlying the effect of cafestol is still unrevealed. The increased level of insulin secretion in our study was accompanied by a significant elevation in insulin biosynthesis, as evident by insulin content and protein expression analysis. Still, more studies are warranted to explain how kahweol induces insulin expression. Additionally, the observed increase in glucose uptake may be attributed to the ability of kahweol to activate Adenosine 5‘monophosphate-activated protein kinase (AMPK), which is considered a central modulator of glucose metabolism [10].

Kahweol has been shown to protect DNA from oxidative stress by inducing HMOX1 to control intracellular ROS levels in human neuroblastoma cells [20]. Kahweol was also praised for its ability to inhibit inducible nitric oxide synthase and macrophage cyclooxygenase-2 expression, as well as its modulator effect on NF kappa-β expression [21]. The current study used STZ, a commonly used chemical in experimental diabetes models, to induce pancreatic β-cell injury [22]. Pancreatic β-cells uptake STZ via Glut2 receptors, resulting in increased oxidative stress due to NO release and ROS production [22]. Pancreatic β-cells are typically prone to oxidative stress because of their relatively low expression of antioxidant enzymes [23]. Indeed, our data showed that INS-1 cells treated with STZ exhibited a dramatic increase in ROS and antioxidant enzymes. HMOX-1, while pre-incubating INS-1 cells with kahweol, reversed the effect of STZ in a concentration-dependent manner. This finding is consistent with previous research indicating that antioxidants may positively impact INS-1 cells [3].

Pre-treatment of INS-1 cells with kahweol effectively inhibited STZ-induced damage, implying that its enhancement of INS-1 cell viability and functionality may be attributed to its apoptotic inhibition. It has been reported that apoptosis induced through STZ in pancreatic β-cells is mediated by ROS [23,24]. This increase in ROS has also been linked to NF-κB activation, a transcription factor that regulates various genes expression associated with cell apoptosis, inflammation, and immunity [25]. The inactivated form of NF-κB is commonly found in the cytoplasm linked with its inhibitor “IκB” to prevent the localization of NF-κB into the nucleus, which is essential for its activity. Among other stimuli, ROS phosphorylates IB kinase, which causes it to dissociate from NF-κB and translocate to the nucleus, promoting transcription by binding to target genes [25]. 

INS-1 cells exposure to STZ significantly induced the expression of NF-κB, while pre-incubation with kahweol effectively reduced the expression. On the contrary, BCL-2, one of the genes regulated by NF-κB, exhibits an anti-apoptotic effect via different mechanisms such as pro-caspases sequestering or inhibiting the release of mitochondrial apoptogenic factors into the cytoplasm [26]. Altogether, these findings suggest that kahweol’s apoptosis reduction could be mediated by NF-κB and consequent regulation of the expression of BCL-2 family protein. Furthermore, these findings concur with the previous studies conducted on STZ-induced diabetic rats [27,28]. 

In addition, STZ-treatment reduced relative p-AKT and BCL-2 protein expression in INS-1 cells, while kahweol significantly upregulated them. These results agree with the previous reports showing that ROS decreased Akt phosphorylation through inhibition of PI3K/AKT pathway [29]. Furthermore, reduced Akt phosphorylation decreases BCL-2, which is an anti-apoptotic protein and one of the downstream molecules of Akt in the PI3K/Akt regulatory pathway [29]. 

It is worth mentioning that reduced expression of numerous essential antioxidant genes, such as GPX1, Sod1–2, and catalase, may contribute to β-cells vulnerability to oxidative stress [30]. On the other hand, other antioxidant genes either exhibit strong expression in β-cells or are noticeably upregulated when oxidative stress is induced (e.g., *Hmox1*, *ID1*, and *ID3*) [31,32]. This study showed that STZ treatment upregulated the expression of antioxidant proteins HMOX1 in addition to ID1 and 3. Our results agree with another study showing an overproduction in HMOX1, ID1, and ID3 in the diabetic mouse model and in vitro H_2_O_2_ induced oxidative stress in MIN6 β-cells [31]. 

In sum, our study demonstrated that kahweol has a protective effect against STZ induced damage in pancreatic β-cells. The effect is possibly mediated through the NF-κB/BCL-2 pathway and pAKT/BCL-2 pathway, alongside affecting the expression of antioxidant proteins such as ID1, ID3, and HMOX1.

## 4. Materials and Methods

### 4.1. Culturing of INS-1 Cell Line 

The clonal rat INS-1 (832/13) cells (a gift from Dr. C. Newgard; Duke University, North Carolina, USA) were maintained in a complete RPMI 1640 medium as previously described [33,34].

### 4.2. Cell Viability Assay

A 20 × 10^4^ INS-1 cells were seeded in a 96-wells plate. Kahweol was purchased from Santa Santa Cruz Biotechnology Inc. (Santa Cruz, CA, USA), and the cells were pre-incubated with different concentrations of kahweol (2.5, 5 and 10 µM) for 24 h followed by STZ treatment (3 mM) for 3 h, as previously described [35]. Untreated cells were used as a negative control. Afterward, cells were incubated with 10 uL of MTT (5 mg/mL) solution (Sigma-Aldrich, St. Louis, MO, USA) and set for 2 h at 37 °C. Formazan crystals were then dissolved in dimethyl sulfoxide (DMSO), and absorbance at 570 nm was detected using a microplate reader. The average 570 nm absorbance values were used to calculate the percentage of cell viability based on the following formula: % cell viability = (OD 570 nm of sample/OD 570 nm of control) × 100.

### 4.3. Apoptosis Assay

Treated cells were washed with buffer saline, followed by resuspension in 500 μL of Annexin-V Binding Buffer (BD Biosciences, CA, USA). A total 5 μL of Annexin V-FITC and PI were used to stain treated cells for 10 min in the dark, followed by analysis using BD FACS Aria III flow cytometer (BD Biosciences).

### 4.4. Insulin Secretion Assays

Glucose–stimulated insulin secretion (GSIS) assay was performed 24 h post-treatment. Briefly, cells were washed twice with secretion assay buffer (SAB) (pH 7.2, 114 m MNaCl, 4.7 m MKCl, 1.2 mM KH2PO_4_, 1.16 mM MgSO_4_, 20 mM HEPES, 2.5 mM CaCl_2_, 25.5 mM NaHCO_3_ and 0.2% bovine serum albumin) containing 2.8 mM glucose, then normalized for 2 h using the same buffer. Next, cells were incubated in 1 mL SAB containing either 2.8 mM glucose or 16.7 mM glucose for 1 h, as previously described [32,33]. Secreted insulin was determined using a rat insulin ELISA kit (Mercodia, Sweden). Insulin content was assayed by extracting total protein using M-PER mammalian protein extraction reagent. Extracted proteins were quantified by Pierce BCA protein assay (Thermo Fisher Scientific, MA, USA), and insulin content (diluted 1:250) was determined using a rat insulin ELISA kit (Mercodia, Sweden) then normalized to the total protein amount.

### 4.5. Glucose Uptake Assay

Glucose uptake was evaluated using a 2-NBDG glucose uptake assay kit (Invitrogen #N13195). The test was performed according to the manufacturer’s instructions. Briefly, 2-NBDG was added over the treated cells and incubated for one hour. Then, cells were harvested and washed with cell-based assay buffer and analyzed immediately with flow cytometry using the FITC detector (Excitation/Emission 485/535 nm).

### 4.6. Intracellular Reactive Oxygen Species (ROS) Measurement

The H_2_O_2_ assay was performed as directed by the manufacturer, as follows. A 20 × 10^4^ of cells per well in a total volume of 100 µL were seeded in a 96-wells plate. The compounds (Kahweol with different concentrations) were added to 96 well-plates in the 80 µL of media for 24 h. After 24 h, the cells were treated with streptozotocin, STZ (3 mM) for 3 h at 37 °C in a 5% CO_2_ incubator. After 3 h of STZ treatment, the H_2_O_2_ substrate solution was added (20 µL), bringing the final volume to 100 µL. Next, the plate was incubated at 37 °C in a 5% CO_2_ incubator for 3 h. After that, 100 µL of the ROS-Glo detection solution was added to each well at the end of the incubation and incubated at room temperature for 20 min. Immediately luminescence was recorded using a plate reader. The average values were used to calculate the relative luminescence unit (RLU).

### 4.7. Western Blot Analysis 

M-PER mammalian protein extraction reagent (Thermo Fisher), containing a protease inhibitor cocktail, was used to extract protein lysate from treated and untreated control cells (Thermo Fisher). SDS-PAGE was used to separate 40 μg of protein lysate, followed by blotting onto a nitrocellulose membrane (Bio-Rad, Hercules, CA, USA). The membrane was blocked using 5% skimmed milk in Tris-buffered saline containing 0.1% Tween 20 (TBST) for 1 h. Subsequently, the blot was incubated overnight at 4 °C with primary antibodies against Insulin (mouse; #8138s, Cell signalling Technology, Danvers, MA, USA), NF-kB (rabbit; #Ab32536; Abcam, Cambridge, UK), BCL-2 (rabbit; #A19693; Abclonal, Wuhan, China), HMOX-1 (rabbit; #ab189491; Abcam), ID1 (rabbit; #ab230679), ID3 (mouse; #ab236505), AKT (#A17909), Phospho-AKT-S473 (#AP0637, from ABclonal technology (Woburn, MA, USA)), or β-actin (#A5441, Sigma-Aldrich, Hamburg, Germany). The membrane was incubated with secondary antibodies (anti-mouse #7076S and anti-rabbit #7074S, Cell signaling Technology, USA) at room temperature for 1 h. ECL substrate kit (Bio-Rad, Hercules, CA, USA) was used to detect chemiluminescence. Bio-Rad Image Lab software (Bio-Rad, Hercules, CA, USA) was used to detect protein bands. Quantification of the bands was done using Image J software. In all experiments, β-actin was used as an endogenous control.

### 4.8. Statistical Analysis

One-way ANOVA nonparametric tests were used for statistical analysis. GraphPad Prism (version 8.0.0 for Windows, GraphPad Software, San Diego, CA, USA,) was used to conduct all statistical analyses. Data within this study are presented as mean ± S.D. Differences were considered significant at *p* < 0.05.

## 5. Conclusions

In conclusion, our data provide evidence that kahweol has an anti-diabetic effect that could prevent or manage diabetes. Furthermore, results revealed that kahweol enhances glucose uptake and reduces ROS in pancreatic β-cells. Thus, the findings of this study pave the way to consider the potential use of kahweol in preventing and treating T2D. However, it is important to keep in mind that the findings of this study are based on in vitro studies. Therefore, further in vivo and clinical studies are required to confirm our findings.

## Data Availability

Data is contained within the article.

## Sample Availability

Samples of the compounds are not available.
